# Self-reported mental health effects of unemployment on young people in Mdantsane, South Africa: A qualitative study

**DOI:** 10.4102/sajpsychiatry.v32i0.2565

**Published:** 2026-01-12

**Authors:** Nyameka Mdewuka, Nongiwe L. Mhlanga, Sikhumbuzo A. Mabunda, Sibusiso C. Nomatshila, Monwabisi Faleni

**Affiliations:** 1 Department of Public Health, Faculty of Medicine and Health Sciences, Walter Sisulu University, Mthatha, South Africa; 2 School of Population Health, University of New South Wales, Sydney, Australia; 3 The George Institute for Global Health, University of New South Wales, Sydney, Australia

**Keywords:** young people, unemployment, Mdantsane, mental health, social isolation, worthlessness, depression

## Abstract

**Background:**

South Africa has been experiencing a persistently high unemployment rate among young people. This high youth unemployment is a stressor to young people, which may culminate in mental health issues.

**Aim:**

The study aimed to describe the self-reported mental health effects of unemployment among young people in Mdantsane township, Eastern Cape province.

**Setting:**

The study was conducted in Mdantsane township, at the Lingomso Youth Centre in the Eastern Cape province, South Africa.

**Methods:**

A descriptive qualitative approach was used. Participants were recruited purposively until data saturation. Data were collected through individual face-to-face interviews. Thematic analysis was conducted using a six-step approach.

**Results:**

The sample comprised 19 young people aged between 19 years and 29 years, and most were men. Two themes emerged: theme one was negative effects, which were characterised by substance use, feelings of worthlessness, stress and anxiety, masking of personality, and social isolation; theme two was that social support buffered the potential adverse effects of unemployment, and this was supported by interdependent role relationships that protected individuals from the effects of unemployment.

**Conclusion:**

There is a need to recognise mental health issues emanating from unemployment, like social isolation, to enable comprehensive, appropriate interventions for young people.

**Contribution:**

This study contributes to understanding mental health issues experienced by unemployed young people. Findings can be used to develop targeted interventions in a large township, such as Mdantsane, in South Africa.

## Introduction

One of the main problems young people face in South Africa is persistent unemployment. By the end of April 2024, almost half (45.5%) of young people between the ages of 16 years and 35 years were unemployed,^1^ while from 2014 to 2024, youth (15–24 years) unemployment constantly fluctuated above 40%.^2^ The Eastern Cape province and KwaZulu-Natal provinces have the lowest rates of young people absorbed into the labour force.^1^ Unemployment among young people is often associated with undesirable mental health effects,^3^ manifested through alcohol and drug use disorders, stress and anxiety disorders, mood disorders and affective disorders.^4^

Therefore, this study sought to describe the self-reported mental health effects of unemployment among young people in Mdantsane township, in the Eastern Cape province of South Africa.

Young people transition from schooling to adulthood and are expected to be employed and assume social and financial responsibilities.^5^ However, the lack of work experience, poor job-seeking skills and mismatch of skills and job requirements among young people make it challenging for them to gain employment compared to older people.^6^ Unemployment may affect mental health through three main pathways: firstly, it is associated with stress because of the day-to-day lack of structure and the stigma of being unemployed. Secondly, the associated financial hardships and potential future reduced earnings are a source of stress. Thirdly, the stress is associated with the social insecurity of not meeting the requirements for securing employment.^7^ The third pathway is characterised by the hopelessness of not finding employment as the duration of unemployment lengthens.^6^ These pathways may increase the risk of mental health issues like stress, anxiety, depressive symptoms, feelings of hopelessness, social isolation, drug and alcohol misuse, and increased screen time.^8,9,10^

In South Africa, corresponding to the high unemployment rates, there has also been a concerning increase in substance use among young people. A study conducted in KwaZulu-Natal province found that common mental health symptoms among young men included worthlessness and substance and/or alcohol abuse, with at least one in four young men contemplating suicide.^11^ Similarly, other South African studies note the association between adverse mental health effects and unemployment. In Soweto (Gauteng province) and Durban (KwaZulu-Natal province), 43.3% of young women had depressive symptoms, and these symptoms were associated with at least 8 h of smartphone use.^12^ Posel et al. also found that employment during the coronavirus disease 2019 (COVID-19) pandemic was significantly associated with lower levels of depression compared to unemployment.^13^

A Swedish study also provides insight into the association between mental well-being and unemployment by highlighting that there are likely to be fewer effects on physical and mental health when unemployment is high, as there is less stigmatisation.^4^ However, the high rates of unemployment may create a sense of hopelessness about ever finding a job, which can cause long-term mental health problems.^4^ Contrarily, unemployment may not affect mental well-being, as individuals have several support systems that offer protection from the problems associated with unemployment.^14^ For example, families can provide emotional and financial support, and social protective measures can be used to address unemployment in the macro-environment.^14^ At the macro-environment, the South African government introduced the special COVID-19 Social Relief of Distress (SRD) Grant in March 2020 for unemployed people aged between 18 years and 59 years without any other grant.^15^ The SRD grant has been turned into a permanent grant for the unemployed.^15^ Notwithstanding the resources and social support available for unemployed people, a systematic review, with most studies drawn from Europe, concluded that 91.4% of 294 studies showed a positive association between high unemployment rates and mental health illnesses of anxiety, mood disorders and suicidal behaviour.^14^ Although there is a positive association, the adverse effects of mental health on unemployment are often buffered by social support and education.^14^

Considering the long period of high unemployment rates in South Africa, the study used Roy’s adaptation model as a theoretical framework. Roy’s adaptation model assumes that people are generally affirmed by purposefulness, which adds value and meaning to life, and this is augmented by activity and creativity.^16^ A person’s ability to cope manifests in four ways, showing responses and interactions with the environment.^16^ The first physical or physiologic mode relates to how a person interacts with the environment through bodily systems. The second is the self-concept, which relates to the behaviour directed from knowing who one is at a given time and how people perceive one as a person. The third is role function, which refers to how one copes because of one’s societal position. The fourth is the interdependent role relationship, which refers to giving love, respect and value, or fostering relational integrity at a group level. The goal of healthcare, as assumed by Roy’s adaptation model, is to promote adaptation, and health should reflect an integrated whole where there is mutuality between the individual and the environment.^16^

Although there is evidence that unemployment may affect mental well-being among young people, to the best of the researchers’ knowledge, there is a paucity of evidence from South Africa, more so from Mdantsane. Additionally, Posel et al.^13^ acknowledge that few studies describe the increased risk of adverse mental health effects of unemployment in South Africa.

A similar study in South Africa described the issue during the COVID-19 pandemic and included young people and older people (more than 40 years old).^13^ In this regard, this study seeks to close a population gap where there is an underrepresentation of young people from Mdantsane and highlight the issue after the COVID-19 pandemic. Therefore, this study sought to describe the self-reported mental health effects of unemployment among young people in Mdantsane township.

## Research methods and design

A qualitative approach with a descriptive design was used. The qualitative approach was chosen as it enabled the collection of in-depth descriptions of the effects of unemployment on young people’s mental health.

### Study setting

The study was conducted in Mdantsane, the second largest township in South Africa, second to Soweto. Mdantsane is in the Eastern Cape province and forms part of the Buffalo City Metropolitan municipality.^17^ The township’s population is predominantly black African, and according to the 2022 Census data, the dependency ratio is 48.3%.^18^

### Population and sampling

The study population was young people aged between 19 years and 35 years who were unemployed or not in school. The study purposively recruited participants who visited the Lingomso Youth Centre.

Potential participants were approached by the researcher, Nyameka Mdewuka (NM), and asked screening questions about whether they were employed or in any training at the time. Those potentially eligible were informed about the study and were recruited if they were willing to participate. The sample size was determined by data saturation, which occurred when information was repeated after participants 17 to 19.

### Data collection

Semi-structured interviews were used for data collection. Individual face-to-face interviews were conducted with the participants. Each interview lasted about 30 min, and data were collected in January 2024. The researcher, NM, whose highest qualification is a postgraduate diploma in health promotion, conducted the interviews in English and isiXhosa. The researcher, NM was trained in data collection and research methods. The data were collected at the Lingomso Youth Centre in Mdantsane. All interviews were recorded after consent to record was obtained from the participants. During the interviews, field notes were taken to record non-verbal cues.

### Data analysis

Data were analysed using Braun and Clarke’s six steps of reflexive thematic analysis.^19^ Two researchers, Nongiwe L. Mhlanga (NM) and Monwabisi Faleni (MF), analysed data independently. The recorded interviews were listened to, read and transcribed for familiarisation. Four transcripts (two for each researcher) were initially selected, and key concepts were identified across the transcripts to develop a codebook. At this stage, the two researchers discussed the identified codes and refined them to develop the codebook. These codes were then condensed into meaningful sentences and phrases to form themes. These developed themes were then reviewed by examining the rest of the transcribed data and subsuming it under the themes developed. The themes were then labelled, and findings were written in narrative format.

Throughout the data collection and analysis process, researchers practised reflexivity to minimise bias. This was ensured by discussing familiarity with the issues of mental health and unemployment throughout. The three researchers, NM, NLM and MF, consciously reflected on their past experiences as young people who had also sought employment. The analysts also avoided paraphrasing transcribed texts so that participants’ experiences could be reflected.

### Ensuring rigour

The study used Lincoln and Guba’s (1988) framework to ensure trustworthiness, which includes four criteria: credibility, transferability, dependability and confirmability.^20^ Credibility was ensured by audio recording interviews; member checking whereby participants checked the transcribed data to ensure it was reflective of what they shared; taking comprehensive field notes; and two researchers conducting data analysis. Documentation of the research procedures and decisions was carried out to ensure dependability. Confirmability was ensured by peer debriefing, whereby an independent researcher, MF, reviewed the study processes continuously, including during and after the interviews, and during and after the data analysis. The peer debriefing ensured that the researchers’ bias was minimised. Transferability was ensured by detailing the study setting.

### Ethical considerations

Ethical approval to conduct the study was granted by the Walter Sisulu University Health Sciences Research Ethics Committee (approval number 111/2023). Participants individually consented in writing to participate.

Participants’ right to confidentiality was protected by using pseudonyms and not including personal identification details that could trace the data to the participants. The researchers were cognisant of the sensitivity of unemployment issues and made prior referral arrangements with the local primary healthcare facility for further support should the need arise, and such a referral was made for two participants.

## Results

The sample comprised 19 participants whose ages ranged from 19 years to 29 years, with an average age of 24 years (standard deviation [s.d.] = ± 3.55). Most (57.9%, *n* = 11) participants were men. Most (63.2%, *n* = 12) participants’ highest educational attainment was matric. The participants’ duration of unemployment ranged from less than 1 year to 4 years, with some (15.8%, *n* = 3) participants noting that they were occasionally engaged in employment, while one (5.3%) did not disclose their duration of unemployment. Concerning the participants’ marital status, most were single (94.7%, *n* = 18). Table 1 shows the participants’ demographic characteristics.

**TABLE 1:** Participant demographic characteristics.

Pseudonym	Age (years)	Gender	Highest educational level	Duration of unemployment in years	Marital status
P1	19	Female	Matric	1	Single
P2	19	Female	Matric	1	Single
P3	26	Male	Matric	4	Single
P4	21	Male	Matric	2	Single
P5	23	Female	Tertiary qualification	1 (occasionally engaged in short-term employment)	Single
P6	22	Male	Tertiary qualification	1	Single
P7	28	Female	Matric	3	Single
P8	21	Female	Matric	Less than 1 year	Single
P9	20	Female	Matric	1	Single
P10	27	Male	Matric	3	Single
P11	29	Male	Matric	Currently unemployed for less than 1 year, but occasionally engaged in short-term employment.	Single
P12	25	Male	Tertiary qualification (diploma in finance)	Uncertain (occasionally engaged in short-term employment)	Single
P13	29	Male	Tertiary qualification (wholesale and retail)	Undisclosed	Single
P14	26	Male	Matric	3	Single
P15	20	Female	Matric	1	Single
P16	20	Male	Matric	Undisclosed	Single
P17	25	Male	Tertiary qualification (studied at SSIPA)	1	Single
P18	27	Male	Tertiary (Mdantsane TVET)	2	Single
P19	29	Female	Matric	4	Married

SSIPA, Sonwa Sakuba Institute for Performing Arts; TVET, Technical and Vocational Education Training.

## Themes

Two main themes emerged: the first theme was negative mental health effects, which was supported by three subthemes. The first subtheme was psychological effects; this was supported by two categories: stress and anxiety, and substance use. The second subtheme was the effect on the self-concept, supported by feelings of worthlessness and masking of character. The third subtheme was the effect on role function, supported by the category of social isolation. The second theme was that the potentially negative effects of unemployment were buffered by social support. The second theme was supported by the subtheme, interdependent roles of young people, which buffer potential adverse effects of unemployment. Two categories supported this subtheme: social interactions are useful, and resources in the physical environment buffer the effects of unemployment.

### Theme 1: Negative effects on self-reported mental well-being of participants

Participants shared how unemployment had negative effects on their mental well-being. These negative effects manifested through psychological effects, young people’s self-concept, and their role function.

#### Subtheme 1.1: Psychological effects of unemployment on mental health

The effects of unemployment on psychological well-being were described by the participants. These effects were stress, anxiety and substance use.

*Category 1.1: Stress and anxiety*: Participants described the stress and anxiety from unemployment. Stress was associated with mental health issues like social isolation and feelings of worthlessness and self-doubt. The quote from P9 illustrates the problems of stress and worthlessness:

‘Unemployment does lead to stress and anxiety in the sense that, as young people, we stay in one place, which leads us to think we are good for nothing.’ (P9, 20-year-old single woman)

From the participants’ shared experiences, stress and anxiety because of unemployment emanated from a lack of material resources to care for themselves and their families, which resonates with the expectation that young people should assume financial responsibilities as they transition to adulthood:

‘I become stressed because I cannot even provide for my family, which results in self-doubt.’ (P12, 25-year-old single man)

‘The fact that I can’t take care of myself stresses me a lot.’ (P5, 23-year-old single woman)

‘My dream was to be independent and try to provide for my family, but being unemployed made me unable to achieve my dreams, so I am stressed.’ (P4, 21-year-old single man)

One participant acknowledged that there were many unemployed young people; however, they were all stressed despite the problem being widespread:

‘Unemployment has led to a higher stress level, and even in our community, we are having a lot of unemployed youth …Too much pressure from society can bring stress and depression.’ (P13, 29-year-old single man)

*Category 1.2: Substance use*: Stress from unemployment was also associated with substance use. Participants P14 and P18 described the use of dagga and Nyaope because of the problems related to unemployment, noting how this was illegal but would help them forget the problem:

‘So, I end up smoking Nyaope and committing a crime.’ (P14, 26-year-old single man)

‘I smoke dagga, and it keeps me dizzy, and I can forget about my problems.’(P18, 27-year-old single man)

Therefore, the quotes above illustrate how the stress associated with unemployment led to maladaptive coping manifested through substance use.

#### Subtheme 1.2: Effects on the self-concept

The second subtheme illustrated the effects of unemployment on young people’s self-concept. Self-concept is built from a person’s internal perception of themselves. These effects are manifested through feelings of worthlessness and masking of one’s true personality.

*Category 1.2.1: Feelings of worthlessness and low self-esteem*: Participants P1, P5, P12 and P16 shared that unemployment made them feel worthless, characterised by low self-esteem, feeling like nothing, and having no purpose in their lives:

‘I have low self-esteem and am scared to express myself when I’m with other people, and it stresses me.’ (P5, 23-year-old single woman)

‘I sometimes don’t avail myself to people and feel like I am nothing.’ (P12, 25-year-old single man)

In addition, worthlessness was felt because of a lack of purpose in life and not being able to attain one’s goals:

‘I cannot achieve my goals, and I can’t pursue my dreams, which leads me to think I’m worthless and have lost hope … I am worthless because I can’t even support my struggling family.’ (P1, 19-year-old single woman)

‘I just sit around and do nothing at home. So, I feel worthless.’ (P16, 20-year-old single man)

Participants shared how they received support from family, friends and the government; however, they still felt worthless. P18 acknowledged the government SRD grant of R350.00 (USD 18.7), while P9, who was an only child with two adopted sisters, was from a family that was relatively affluent, but they still felt worthless:

‘I just think that I am worthless. I cannot do anything for myself; I only depend on the R350.00 grant.’ (P18, 27-year-old single man)

‘I am the only child at home with two adopted sisters … I feel worthless; I used to think that being unemployed happens to poor people or to those who are not good enough or not very intelligent. I come from a family that can afford, but here I am, unemployed.’ (P9, 20-year-old single woman)

From the quotes above, it was concluded that unemployment made young people feel worthless because of the lack of purpose in life and the lack of financial stability.

*Category 1.2.2: Masking of one’s personality around family and friends*: Another negative coping behaviour was described by P16, who had also highlighted that unemployment made them feel worthless. The participant in this regard added that when they are among family and friends, they mask their real personality and display various characters, noting that this portrayal of various characters had brought out their artistic skills:

‘Yes, I’ve become an artist …I don’t have much choice, so I’ve created these characters to mask the real me.’ (P16, 20-year-old single man)

The participant was probed on whether this was their coping strategy for managing the stress associated with unemployment, and they noted that they only did this around people; however, when they were alone, they also listened to music.

‘When I am alone, I do listen to Adele’s music.’ (P16, 20-year-old single man)

However, overall, the participant noted that they felt trapped by growing up because of the responsibilities expected of them:

‘… because of these responsibilities that come with growing, so much that I even say at times growing is a trap.’ (P16, 20-year-old single man)

#### Subtheme 1.3: Role function

The third negative way unemployment affects mental health is through young people’s role function. The role function is what is expected of someone when they occupy a certain role in society. In this regard, young people are expected to assume financial responsibilities as they transition to adulthood.^5^ However, because of unemployment, young people often isolate themselves as they cannot fulfil their roles.

*Category 1.3.1: Social isolation*: From their shared experiences, all participants lived with family except for P13, who stayed alone and had moved from the Western Cape province to Mdantsane township in search of employment. Notably, P13 also shared that they isolated themselves:

‘Being unemployed leads to stress because there are things that I need and cannot afford, and I end up spending most of my time alone.’ (P13, 29-year-old single man)

Some participants, P2, P4 and P14, shared that they had attended schools and higher education institutions where they had made friends within the community. However, they preferred isolating themselves despite having family and friends around them. P14 explained how they isolated themselves from both family and friends and ended up having depressive symptoms:

‘I spend most of my time in my room as I cannot socialise with others because of my situation. I ended up having depression.’ (P14, 26-year-old single man)

Some participants explained how they isolated themselves from their friends who were employed. This isolation was shared from a self-protective viewpoint, as a way of guarding against jealousy, feeling insecure, or not wanting pity:

‘Yes, I isolate myself because it makes me insecure, as my other friends can get everything they want, and I can’t get anything I want because I am unemployed.’ (P2, 19-year-old single woman)

‘I do not want my peers to feel sorry for me, so I like to be alone.’ (P4, 21-year-old single man)

‘Sometimes isolating yourself keeps one from hearing or seeing our friends who are working, and by isolating, you can prevent yourself from being jealous towards others’ progress. This affects health and well-being as we start involving ourselves or having suicidal thoughts simply because we feel that life is unfair to us.’ (P9, 20-year-old single woman)

In summary, the unemployed young people shunned socialisation to protect their emotional and mental well-being, especially among social networks who were employed.

### Theme 2: Social support buffers the potential adverse effects of unemployment

The second main theme developed was that social support buffers the potential adverse effects of unemployment. This theme was supported by the subtheme interdependent role relationships protect from unemployment, which subsumed two categories:

‘I do not have any health problems…I still have hope that things will eventually work out at the right time. They say you don’t choose a job; you take whatever comes, and what is best for you will come later.’ (P11, 29-year-old single man)

‘There are times when I wish I were employed so that I can financially support myself, but I just usually accept the situation and hope to overcome it.’ (P8, 21-year-old single woman)

#### Subtheme 2.1: Interdependent role relationships buffer the potential adverse effects of unemployment

The giving and receiving of love with significant others form a social support system that can buffer the adverse effects of unemployment. Participants indicated they had family and friends who provided social support.

*Category 2.1.1: Social interaction among unemployed young people is useful*: Some participants, P6, P8, and P10, highlighted how they interacted with friends to gain information on seeking employment and/or simply to have an opportunity to forget the stress associated with unemployment:

‘Being around people greatly helps me because I forget about being unemployed.’ (P6, 22-year-old single woman)

‘I mix myself with working people so that they can give me advice on how to find a job or what to do.’ (P8, 21-year-old single woman)

‘I have friends who always encourage me, and I also think positively all the time.’ (P10, 27-year-old single man)

Participants also acknowledged the role played by family members in ensuring mental well-being. Participant P19, who was married and had children, shared that they would not isolate themselves and would interact with their children. P10 and P8 also interacted with friends and acknowledged parental support:

‘I was raised by both parents: mom and dad. I grew up in a loving, happy, and supportive family.’ (P8, 21-year-old single woman)

‘My parents are very supportive, and they encourage me to believe in myself.’ (P10, 27-year-old single man)

‘Even if I wanted to isolate myself, I would not. I have children that I must take care of, and they demand a lot of my time.’ (P19, 29-year-old woman, married)

From the participants’ responses, it was concluded that friends were helpful as they provided information on seeking employment, while family encouraged young people, thereby ensuring potential adverse effects of unemployment are buffered.

*Category 2.1.2: Resources for young people in the physical environment provide support*: Participants described how communal resources like the Lingomso Youth Centre supported them against the potential adverse effects of unemployment. People create group identity through association with such resources in the physical environment. This, in turn, had an effect at the individual level by providing support against the potentially adverse impact of unemployment:

‘I go to Lingomso Youth Centre. Yes, they don’t employ people, but they give you the opportunity to expose yourself to things that might benefit you one day.’ (P8, 21-year-old single woman)

‘Yes, there is, and it advises about coping with stress. The place is Lingomso Youth Centre.’ (P11, 29-year-old single man)

‘There are a few like Lingomso Youth Centre and my church.’ (P19, 29-year-old woman married)

The support provided by resources in the environment was also shared by participants who reported that they experienced the negative impact of unemployment on mental well-being. Quotes from P5, who described how they were stressed because of unemployment, and P15 support the category:

‘I go to the club at Lingomso Youth Centre to meet with other club members. I have also been guided by coaches at the centre.’ (P5, 23-year-old single woman)

‘Young people of Mdantsane should use the services that are provided by Lingomso Youth Centre.’ (P15, 20-year-old single woman)

The above quotes indicate how young people were supported by community resources like the youth centre and the churches, where they learnt positive coping strategies to manage stress and had opportunities to network to increase their chances of gaining employment, thus buffering the potential adverse effects of unemployment.

## Discussion

The findings revealed that young people experienced negative effects of unemployment on their mental health. These negative effects manifested through stress and anxiety, alcohol and/or substance misuse, feelings of worthlessness, masking of personalities and social isolation. Social support may buffer the potential adverse effects of unemployment. The study included young people aged 19–29 years in South Africa. We expected to include participants aged over 29 years, but data saturation was reached before we had recruited any participants aged over 29 years, which may limit our findings to young people aged under 29 years. The sample also comprised more male participants, which may not reflect current trends of unemployment in South Africa, which indicate that more women are unemployed.^21,22^

Regarding the negative effects of unemployment on mental well-being, several studies^8,9,10,12^ have also found that unemployment among youths is associated with poor mental health. A systematic review conducted by Virgolino et al.,^14^ which drew most studies from Europe, noted that the most common mental health issues associated with unemployment manifest through anxiety and mood disorders. This study confirms the stress and anxiety experienced among unemployed youths. Furthermore, Wilson and Finch^7^ explain that the pathways in the development of mental health issues associated with unemployment emanate from stress associated with poverty. This study affirms this pathway, as participants noted that their source of stress was a lack of financial resources.

The study found that one of the negative effects of unemployment on mental health was social isolation. Mokona et al.^9^ in the Ethiopian study noted that depressive symptoms are associated with social isolation among unemployed young people. In this study, the issue of unemployment, social isolation, and depression was described by P14, who noted that they isolate themselves and end up being depressed. Therefore, taking note that the Centre for Epidemiological Studies Depression Scale lists loneliness as one of the indicators of depression,^23^ when participants discussed the issue of social isolation, this may have indicated underlying depressive symptoms. As such, this may require facilitation for further mental health assessment and subsequent intervention. In South Africa, a longitudinal study also confirms that depressive symptoms are associated with unemployment or transitioning from employment to unemployment.^24^

In this study, the participants described low self-esteem issues related to the lack of purpose and income as the source of feelings of worthlessness. A rural KwaZulu-Natal study also confirmed that feelings of worthlessness are associated with depression among young people.^11^

This study also confirmed findings from a Durban (South Africa) study, which found that there is a significant inverse relationship between depression and being ashamed of the lack of work among men.^25^ The issue of masking one’s personality resonates with the issue of camouflaging, where characteristics are concealed in social situations.^26^ Camouflaging is commonly associated with autism and is a means to control the impression that others have of a person.^26^

Alternatively, the issue of masking one’s personality can also be indicative of broader stigma associated with unemployment, as described in an Australian study, whereby the unemployed try to cover their pain by presenting positive public identities.^27^ Notwithstanding the different explanations, further research may be needed to explore the issue in South Africa.

In South Africa, the issue of substance and alcohol misuse is pertinent among young people, with studies in Gauteng and Durban highlighting the issue.^11,12^ This study confirms the problem of substance misuse among young people who indicated that stress because of unemployment led to substance and alcohol misuse. Further confirming the association between substance use and unemployment, a cross-sectional study also conducted in the Eastern Cape found that male gender and younger age were significantly associated with the likelihood of substance use.^28^

Young people in this study described the buffering role of social networks. This finding is explained by Virgolino et al.^14^ who highlighted that unemployment may not affect mental health when social support is available. However, the finding still contradicts several studies^4,8,9,10^ which note that unemployment has negative mental health effects. This is not unexpected; an explanation by Thern et al.^4^ notes that during periods of unemployment crises, there could be less impact on mental health as the stressor is external and experienced by many people.

Unemployment in South Africa has been high since 2014, which may explain the externalisation of the problem. The previous systematic review conducted by Virgolino et al.^14^ further highlighted the mitigating effects of resources in the physical environment, which the study participants acknowledged.

## Conclusion

Unemployment in South Africa remains pervasive, affecting young people’s mental well-being as they transition to adulthood. This study provided insight into young people’s self-reported mental health effects from unemployment in Mdanstane. The study findings show that it is critical to provide mental health services as part of the services offered in community youth centres in South Africa. The study recommends additional studies that focus on the nexus between unemployment and substance use, given the persistently high unemployment rates in South Africa and the high prevalence of substance use among young people. This study had several limitations; firstly, the exclusion of young people’s families, who could also provide in-depth insight into mental health issues affecting young people. This view is further strengthened by the fact that several participants referred to family. Secondly, the small sample size and exclusion of those aged more than 35 years limit the generalisability of the study findings.

Lastly, there was potential researcher bias as the NM who collected data was also a young person in the same age category as the participants. Notwithstanding this limitation, the study’s strength was the selection of participants from a youth centre who were able to provide insights that provided theme saturation.
